# Virtual screening of the inhibitors targeting at the viral protein 40 of Ebola virus

**DOI:** 10.1186/s40249-016-0105-1

**Published:** 2016-02-17

**Authors:** V. Karthick, N. Nagasundaram, C. George Priya Doss, Chiranjib Chakraborty, R. Siva, Aiping Lu, Ge Zhang, Hailong Zhu

**Affiliations:** School of Chinese Medicine, Hong Kong Baptist University, Kowloon Tong, Hong Kong; Department of Integrative Biology, School of Biosciences and Technology, VIT University, Vellore, Tamil Nadu India; Department of Bioinformatics, School of Computer and Information Sciences, Galgotias University, Noida, India; Department of Biotechnology, School of Biosciences and Technology, VIT University, Vellore, Tamil Nadu India

**Keywords:** Ebola, VP40, Traditional Chinese Medicine Database, Molecular docking, Molecular dynamics

## Abstract

**Background:**

The Ebola virus is highly pathogenic and destructive to humans and other primates. The Ebola virus encodes viral protein 40 (VP40), which is highly expressed and regulates the assembly and release of viral particles in the host cell. Because VP40 plays a prominent role in the life cycle of the Ebola virus, it is considered as a key target for antiviral treatment. However, there is currently no FDA-approved drug for treating Ebola virus infection, resulting in an urgent need to develop effective antiviral inhibitors that display good safety profiles in a short duration.

**Methods:**

This study aimed to screen the effective lead candidate against Ebola infection. First, the lead molecules were filtered based on the docking score. Second, Lipinski rule of five and the other drug likeliness properties are predicted to assess the safety profile of the lead candidates. Finally, molecular dynamics simulations was performed to validate the lead compound.

**Results:**

Our results revealed that emodin-8-beta-D-glucoside from the Traditional Chinese Medicine Database (TCMD) represents an active lead candidate that targets the Ebola virus by inhibiting the activity of VP40, and displays good pharmacokinetic properties.

**Conclusion:**

This report will considerably assist in the development of the competitive and robust antiviral agents against Ebola infection.

**Electronic supplementary material:**

The online version of this article (doi:10.1186/s40249-016-0105-1) contains supplementary material, which is available to authorized users.

## Multilingual abstracts

Please see Additional file 1 for translations of the abstract into the six official working languages of the United Nations.

## Background

The Ebola virus is a virulent pathogen that causes haemorrhagic fever in humans and animals, and that leads to a fatality in nearly 90 % of cases within 7–11 days of infection [1]. The Ebola virus has proven to be dangerous in many African countries, thereby contributing to its higher incidence of death [2]. Ebola carries a negative-sense RNA genome containing seven genes that encode seven different structural proteins: VP24, VP30, VP35, VP40, a glycoprotein, a nucleoprotein and a polymerase (L) [3]. In particular, the major matrix protein VP40 appears to be highly expressed in Ebola virus and plays a vital role in the budding of Ebola virus from the plasma membrane [4].

Additionally, VP40 participates in host cell RNA metabolism during the replication process [5, 6]. The crystallographic structure of VP40-RNA reveals that the R-134 and F-125 of VP40 mainly interact with RNA [7]. These interactions play a crucial role in octamer formation and promote the replication of the Ebola virus. Mutational studies have shown that F-125 and R-134 mutations partially reduce RNA binding and completely abolish RNA binding and octamer formation, respectively [8]. It is clearlyevident from the literature that the VP40-RNA binding mechanism is crucial in the process of viral transcription at early stages of infection [9]. These studies strongly suggest that the binding of RNA to VP40 could be the critical factors for the successful life cycle of the Ebola virus. Because VP40 plays a vital role in the Ebola virus life cycle, it is considered as a potential target for the treatment of Ebola virus infection. However, the pharmacological inhibition of VP40 has yet to be studied in detail [6]. Meanwhile, no FDA-licensed drug is currently available for the treatment of this lethal infection [10]. Thus, the screening of small molecules against Ebola virus that target VP40 might shed light on the antiviral treatment of this disease.

To address this issue, the Traditional Chinese Medicine (TCM) Database (TCMD) [11] was used to identify novel, potent lead compounds to combat Ebola infection. At present, Computational approaches plays a vital role in biological research. Virtual screening is one of the most important promising techniques aids in dealing with a large number of lead molecules and ranks them using molecular docking. As of today, many drugs are optimized using computational approaches (Captopril, Dorzolamide, Saquinavir, Zanamivir, Oseltamivir, Aliskiren, Boceprevir, Nolatrexed, TMI-005, LY-517717, Rupintrivir and NVP-AUY922) [12].

Previous studies have showed that the combination of virtual screening with molecular docking and molecular dynamics approaches can successfully identify novel drug-like molecules for the treatment of infectious diseases [13–18]. In this study, we also predicted the oral toxicity (LD50) of the screened lead compounds to determine their side effects and bioavailability using computational methods.

## Methods

### Datasets

The crystal structure of the matrix protein VP40 from the Ebola virus was obtained from RCSB [19]. The corresponding PDB code is 1H2C [7]. The three-dimensional structures of the lead compounds were retrieved from the TCMD. The three-dimensional structures of the target proteins were energy-minimized using the GROMACS package, version 4.6.3 [20, 21] adopting the GROMOS43a1 force field parameters before performing the docking analysis. Additional file 2: Figure S1 depicts the overall flow chart of the present study.

### Virtual screening

Virtual screening is an essential technique that is of immense importance to the field of drug discovery. This method is considered as an alternative approach to experimental screening and has produced an increased success rate in the drug discovery process [22, 23]. Because there is no FDA-approved drug available for treating Ebola virus infection [24–26], we used iScreen to apply a receptor-based virtual screening approach. iScreen is a compact web server for TCM docking and virtual screening [27]. iScreen utilizes the PLANTS package, which is based on colony optimization, as a docking algorithm [28]. This algorithm can efficiently rank the potential lead candidates. The three-dimensional structure of VP40 was used as the input for identifying the optimal lead compounds from TCMD. The RNA-VP40 binding site residues are used for virtual screening of lead compounds.

### Molecular docking analysis

The top ranked lead compounds were further evaluated via molecular docking analysis using AutoDock 4.2.6 [29]. We have used different docking algorithms and repeated docking to increase the accuracy and reliability of the docking result and to reduce the false positive outcome. The AutoDock tools were used for the addition of charges and polar hydrogens and the adjustment of other parameters. Additionally, Autogrid was used to generate grid maps and spacing [30]. Furthermore, the Lamarckian genetic algorithm (LGA) was employed to perform molecular docking. Each docking experiment consisted of 10 different docking runs, which were set to terminate after 250,000 energy evaluations. The docking calculation included a population size of 150 and a translational step of 0.2 Å, and the docking results were ranked according to the binding free energy and the frequency of the most probable binding site. Also, the energetic contribution of screened lead compounds and VP40 was analysed using PEARLS [31]. Furthermore, the intermolecular interactions of the complexes were analysed.

### Intermolecular interaction analysis

In addition to the binding affinity of the molecules, their inhibitory effect can be determined by analysing their interactions with receptor molecules. In particular, hydrogen bond interactions ensure the stability of drug-receptor complexes. Thus, we use PDBsum [32] and Chimera [33] to analyse the intermolecular interactions.

### Molecular dynamics simulation

The structures of the docked complexes of VP40 with the screened lead compounds were used as the starting point for MD simulations using the GROMACS package, version 4.6.4 [20, 21] adopting the GROMOS43a1 force field parameters. The structures were solvated in a cubic box with a size of 0.9 nm using periodic boundary conditions and the SPC water model [34]. The topology of the lead compounds was generated using the PRODRG server [35]. Subsequently, energy minimization was performed for both complex structures using the steepest descent energy protocol. Furthermore, the systems were equilibrated by performing a position-restrained dynamics simulation (NVT and NPT) at 300 K for 300 ps. Then, the equilibrated structures were subjected to molecular dynamics simulations for 50,000psata constant temperature of 300 K and pressure of 1 atm, and the integration time step was set to 2 fs. The non-bonded list was generated using an Atom-based threshold of 8 Å. Long-range electrostatic interactions were managed using the particle-mesh Ewald algorithm [36]. A 0.9 nm threshold was employed Lennard-Jones interaction. During the simulations, the lengths of all bonds containing hydrogen atoms were constrained utilizing the Lincs algorithm [37]; the trajectory snapshots were stored for structural analysis every picosecond. The RMSD and the hydrogen bonds were analysed using the Gromacs utilities g_rms and g_hbond. Furthermore, the MM-PBSA [38] was calculated to determine the binding free energy between VP40 and the lead compounds.

### ADME analysis and Drug likeliness analysis

Lipinski’s rule of five was used to testthe bioavailability characteristics, such as the absorption, distribution, metabolism and elimination (ADME), of the lead compounds. In the present study, these molecular properties and the drug-likeness of the lead compounds were estimated using the Molsoft program (http://molsoft.com/mprop/).

### Prediction of toxicity risk and oral toxicity (LD50)

We predicted the preclinical oral toxicity (LD50) of the lead compounds using the Osiris Property Explorer (http://www.organic-chemistry.org/prog/peo/) and the ProTox web server [39], respectively. The ProTox web server prediction method is based on the analysis of two-dimensional (2D) similarity to compounds displaying known LD50 values and on the identification of over-represented fragments in toxic compounds. The ProTox server integrates toxicity class prediction using similarity- and fragment-based methods with alerts for possible toxicity targets, thus providing insight into the mechanisms involved in the development of toxicity.

## Results

### Virtual screening and docking analysis

**Fig. 1:**
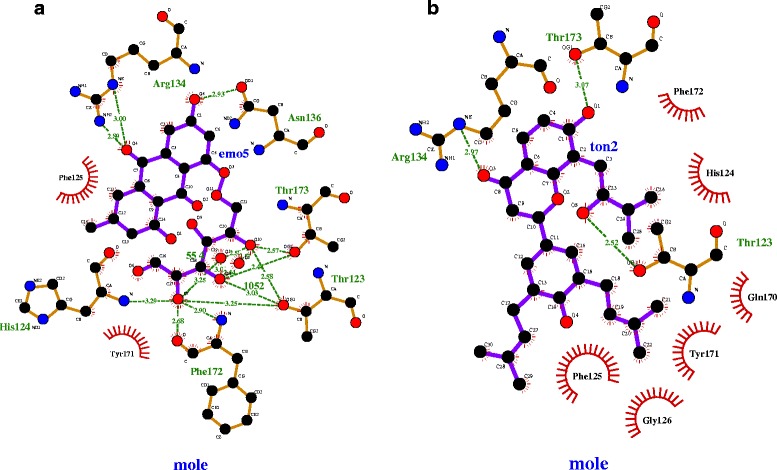
The interaction of compound 1 (**a**) and compound 2 (**b**) with VP40

TCM plays an essential role in the field of medical diagnosis and treatment in East Asia. TCM emphasizes systemic health using medicines originating from natural herbs that exhibit few or no side effects. TCM has been employed in China for thousands of years and remains a very successful treatment modality in the medical field [11]. The TCMD facilitates the virtual screening of TCM compounds. Recently, increasing efforts have been devoted to studying the importance of TCM by isolating bioactive compounds from the medicinal herbs employed in TCM and by examining their activities. Therefore, we employed the TCM database to screen for effective antiviral agents against Ebola virus infection. A total of 200 compounds from the TCM database were screened on the basis of docking score, and the three top-ranked compounds were sorted according to their docking scores. The lists of top 15 compounds and their respective docking scores were shown in Additional file 2: Table S1. The top 3 compounds are selected for the further analysis. The docking scores of compounds 1, 2 and 3 were -84.05, -82.62 and -82.44, respectively (Additional file 2: Table S2). Furthermore, we performed docking analysis using AutoDock to identify the binding affinity between the target and each lead compound. To reduce the inconsistency of the results, we performed repeated docking analyses. Information on the binding site residues of VP40 was collected from the available literature [7]. The docking results indicated that the average binding affinities of compounds 1 and 2 were higher than that of compound 3, as shown in Additional file 2: Table S3. Thus, we performed further analyses on these two lead compounds. The target protein structures with 2 lead molecules in the docked conformation were validated using PROCHECK to check for the steric clashes occurred during docking experiment. The result showed that the 100 % of residues fall in most favoured and allowed regions in both complexes. This shows that both the docked conformations free from steric clashes. Furthermore, the energetic contribution of lead compounds and RNAwas analysed using PEARLS. This analysis confirmed that the compound 1 and compound 2 exhibit better binding interactions with VP40 (Additional file 2: Table S4). PDBsum [32] was used to visualize the interactions of the complex structures. The interactions between VP40 and the lead compounds are shown in Fig. 1. Compounds 1 and 2 maintained 6 and 3 hydrogen bond interactions, respectively (Additional file 2: Table S5).

**Fig. 2:**
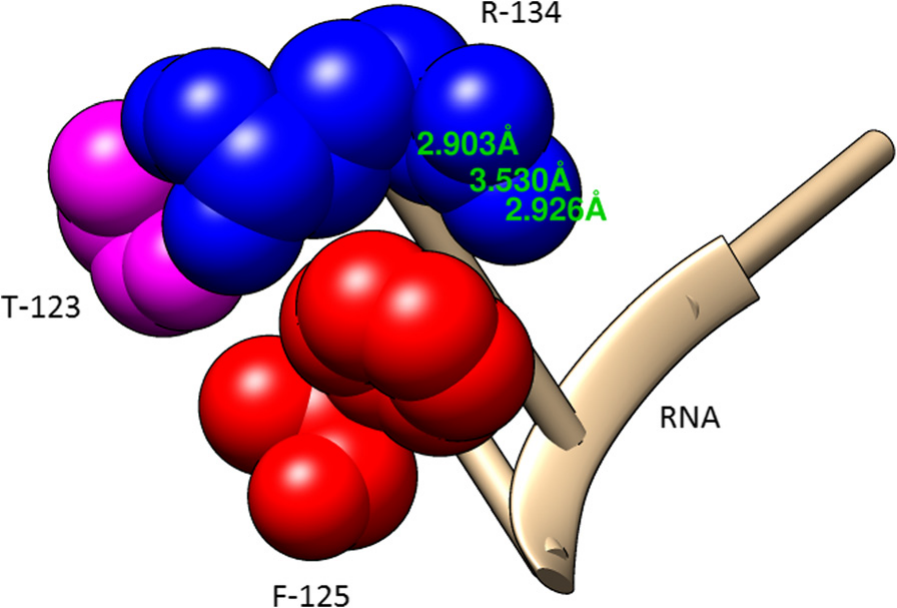
Chimera visualization of the key interacting residues between RNA and VP40 (R-134, F-125 and T-123)

**Fig. 3:**
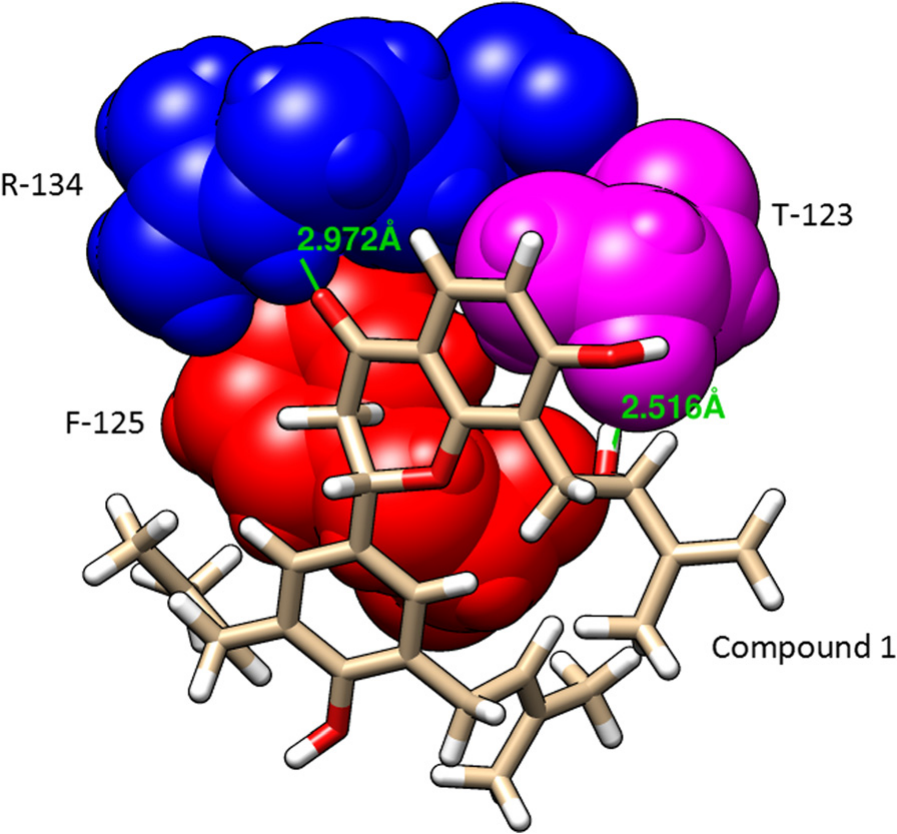
Chimera visualization of the key interacting residues between compound 1 and VP40 (R-134, F-125 and T-123)

**Fig. 4:**
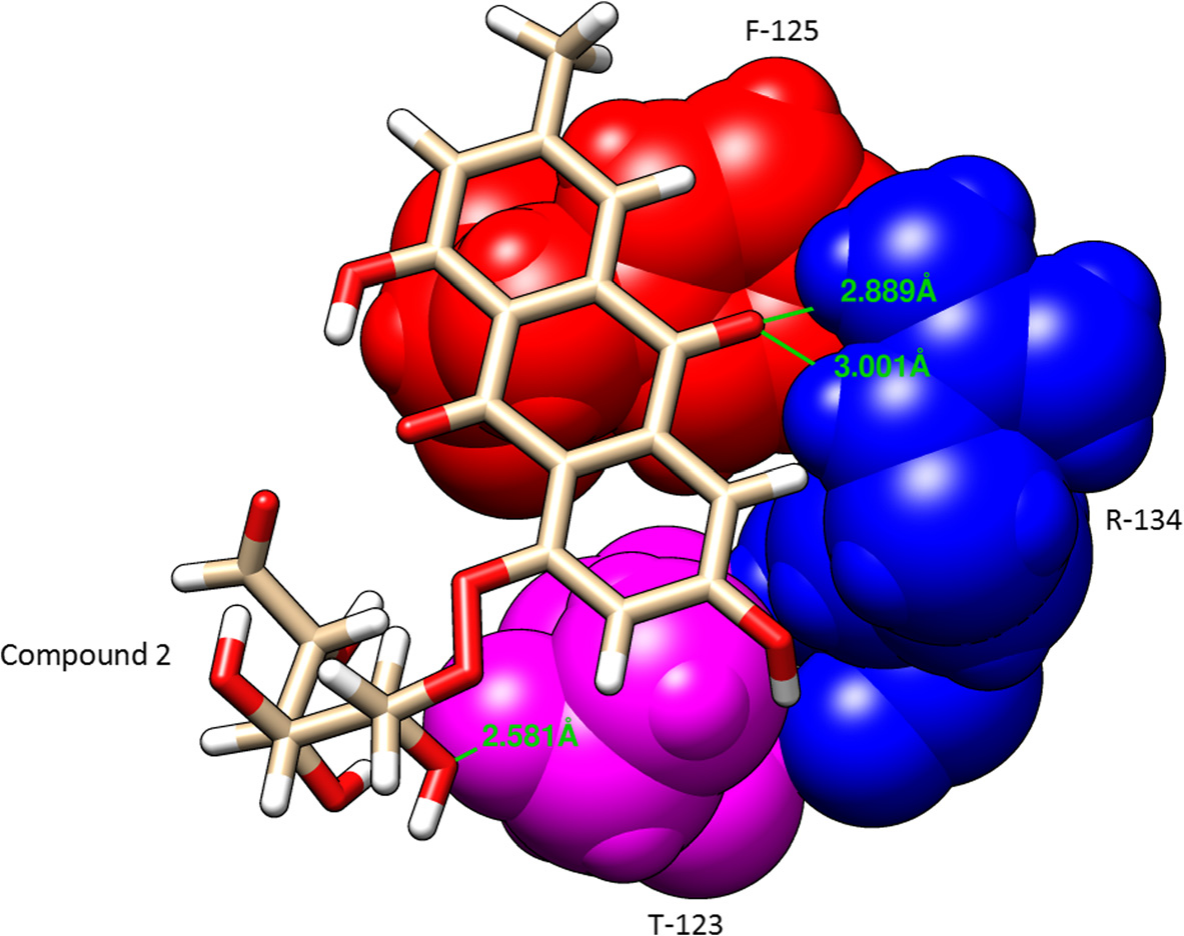
Chimera visualization of the key interacting residues between compound 2 and VP40 (R-134, F-125 and T-123)

Furthermore, it has been clearly indicated in previous studies that two conserved residues, F-125 and R-134, are the most critical residues for RNA binding in the Ebola virus and that R-134 plays a vital role in the replication of this virus [7]. Therefore, we analysed these key interactions between VP40 and RNA using Chimera [32]. The observed interaction between VP40 and RNA indicated that R-134 of VP40 interacts with RNA via a hydrogen bond and forms close contacts with T-123 and F-125 (Fig. 2). Similarly, it is notable that two lead compounds were able to establish a hydrogen bond interaction with R-134 and T-123 and were found to form a close interaction with F-125 (Figs. 3 and 4), which has been demonstrated to be crucial for the inhibition of Ebola virus replication. Furthermore, the critical atoms of lead compounds and RNA forming hydrogen bonds with VP40 are depicted in (Additional file 2: Figures S2, S3 and S4).

### Molecular dynamics simulation

**Fig. 5:**
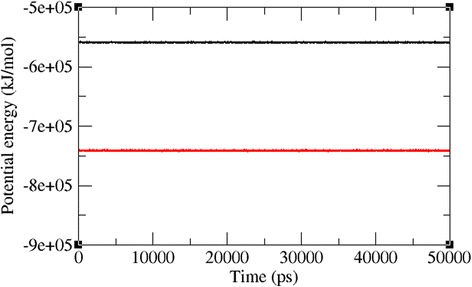
Potential energy variation for the VP40-compound 1 (black) and VP40-compound 2 (red) along the MD simulation

**Fig. 6:**
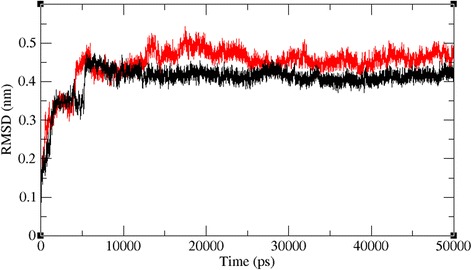
Root mean square deviations correspond to VP40-compound 1 (black) and VP40-compound 2 (red) along the MD simulation

**Fig. 7:**
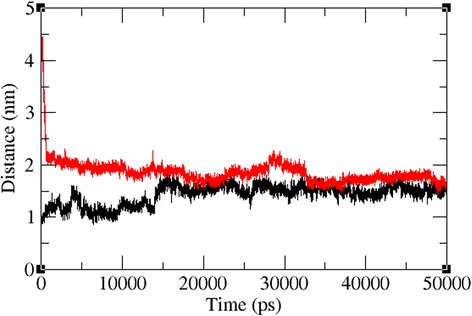
Distance between R-134 of VP40 with compound 1 (black) and compound 2 (red) along the MD simulation

**Fig. 8:**
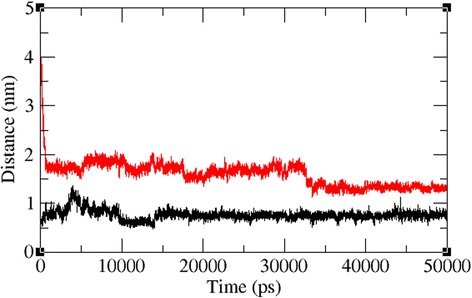
Distance between F-125 of VP40 with compound 1 (black) and compound 2 (red) along the MD simulation

**Fig. 9:**
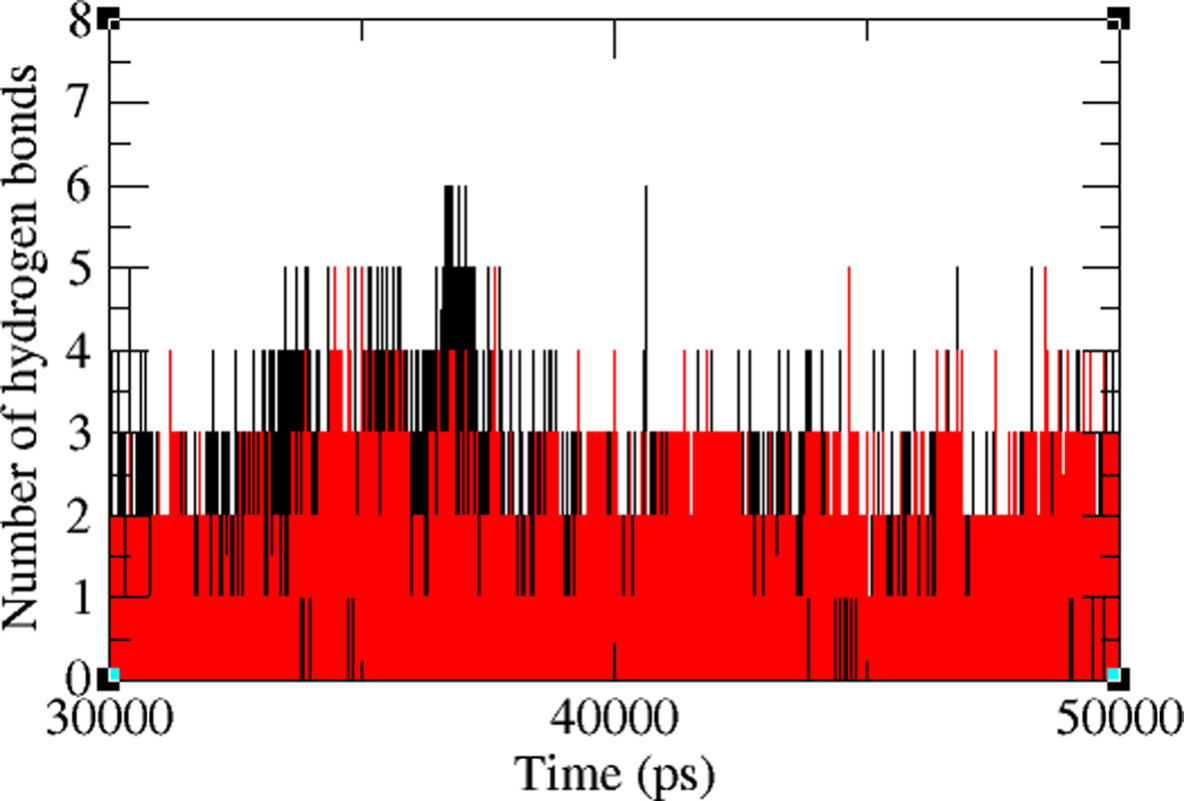
Hydrogen Bonds observed between the protein and ligand along the MD simulation. The symbol coding scheme is as follows: VP40-compound 1 (black) and VP40-compound 2 (red)

The docked complexes of the two lead molecules were used for the molecular dynamics simulation. The potential energy plots obtained from the MD simulation showed that both simulated interactions are stabilized throughout the simulation process (Fig. 5). In addition, root mean square deviation (RMSD) analysis showed that the VP40-compound 1 interaction displayed an RMSD of ~0.45 at 50 ns into the simulation and that the VP40-compound 2 interaction displayed an RMSD of ~0.48 at the end of the simulation. Both complexes displayed convergence at ~30 ns into the simulation, indicating the stability of both simulated interactions (Fig. 6). RMSD results indicate that movement of both compounds are small and thereby showing the strength of these compounds within the binding pocket of the VP40. To further validate the binding strength of these compounds and VP40. We plotted the distance between the lead compounds and crucial binding residues (R-134 and F-125, which illustrates the role in RNA binding and octamer formation. Figures 7 and 8 show that both compounds 1 and 2 were situated a short distance from R-134 and F-125, with minimal variation. This analysis indicated that the both compounds 1 and 2 can competitively bind to this target and may suppress the binding of RNA to VP40 and also restrict the octamer formation which is a key factor for viral replication. Also, it is well known that hydrogen bond contribution of protein-ligand determines the binding strength of the complex. The hydrogen bonds reveal that both the compounds interact with VP40 with a higher number of hydrogen bonds. Figure 9 shows that compound 1 and 2 maintained 5-6 and 3-4 hydrogen bonds, respectively. These analyses lead us to predict the consistent binding strength of the compounds throughout the simulation time.

**Fig. 10:**
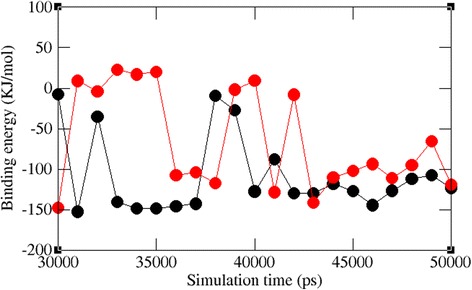
Binding energy observed between the protein and ligand along the MD simulation. The symbol coding scheme is as follows: VP40-compound 1 (black) and VP40-compound 2 red)

Although interaction analysis and hydrogen bond analysis helps in determining the binding of the compounds, binding free energy from molecular dynamics calculation is always crucial in determining the binding affinity of the lead molecules in different time scale. Hence, we calculated the binding free energy between VP40 and the lead compounds using the molecular mechanics-Poisson-Boltzmann surface area (MM-PBSA) approach. As shown in Fig. 10, the average binding energy of compounds 1 and 2 to VP40 ranged from ~ -50 to -150 KJ/mol, indicating a good binding affinity of compound 1 and 2 to VP40. This analysis revealed that the targetable to maintain the tight binding of the compounds during the whole simulation time.

### ADME and drug-likeness analysis

Based on the analysis described above, it was predicted that compounds 1 and 2 are stable and display a high binding affinity to the drug target. Molecular properties such as the partition coefficient (logP), molecular weight (MW), and the number of hydrogen bond acceptors and donors in a molecule are always considered when predicting the bioavailability of the molecule [40]. These molecular properties were used to formulate the “rule of five”^21^. This rule states that most molecules displaying good membrane permeability exhibit a molecular weight ≤500, a calculated octanol–water partition coefficient, logP ≤5, hydrogen bond donor’s ≤5 and hydrogen bond acceptors ≤10 [41]. Therefore, the molecular properties and bioactivity of these lead compounds were predicted using the Molsoft program (http://molsoft.com/mprop/) based on the Lipinski rule of five, which states that an orally active compound should have no more than one violation. The results showed that both compounds 1 and 2 displayed one violation, which is acceptable according to the Lipinski rule of five (Additional file 2: Table S6) [42, 43]. Furthermore, drug-likeness is a key factor that determines the efficacy of the drug in terms of favourable absorption, distribution, metabolism, excretion and toxicological (ADMET) parameters. The drug-likeness values of compounds 1 and 2 were 0.88 and 0.36, respectively, which indicated that 1 displays higher druggability than compound 2.

### Toxicity risks and oral toxicity (LD50) analysis

Drug discovery is a complicated procedure that requires compounds to be highly bioavailable and safe to enter the clinical phase. Toxicity and side effects are the major issues that lead to the failure of a drug during its development. Animal trials are currently the predominant method used to determine the possible toxic effects of drug candidates and cosmetics. *In silico* prediction serves as an alternative approach for simplifying and rationalizing drug development at the preclinical stage, thereby helping to minimize the cost, time, and animals involved [44]. Therefore, we used the Osiris Property Explorer to assess the toxicity risk of the screened lead compounds. The analysis indicated that neither of these lead compounds exerts any mutagenic, tumorigenic or reproductive effects (Additional file 2: Table S7).

Furthermore, we used the Protoxweb server to calculate the LD50 value of the screened lead compounds. Higher the LD50 dose, lower the toxicity of the compound. The predicted oral toxicity of compound 1 was 5000 mg/kg, and the toxicity class is in the range of 5. These results indicate that compound 1 displays a better safety profile than compound 2 (Additional file 2: Table S7).

## Discussion

Ebola infection has become a significant challenge to human life, as Ebola has killed millions of people thus far (http://www.cdc.gov/vhf/ebola/outbreaks/history/distribution-map.html). Various efforts have been introduced to develop effective vaccines against this disease. However, no concrete report has demonstrated the pharmacological inhibition of the Ebola virus. Because the fatality rate of Ebola in humans is increasing each day, there is an urgent need to develop potential drugs at a faster pace. Thus, we adopted a computational approach to support experimental biologists in developing an effective drug in a shorter duration. Virtual screening is a modern technique that is used to prioritize active hits based on their binding affinity to a target. Many successful drug candidates have been developed against various diseases using this technique. In particular, molecular dynamics-based virtual screening is helpful for predicting the quality of screened lead compounds. As TCM, the most reliable source of medications, we employed the TCMD for virtual screening.

In this report, we have computationally identified 2 TCM-based lead candidates, emodin-8-beta-D-glucoside and tonkinochromane_G, as potential inhibitors of Ebola infection. VP40 is a core target for antiviral agents because of its essential role in the replication of the Ebola virus. VP40 binds to RNA, which forms an octameric ring structure to promote the replication of the virus. Interaction analysis showed that RNA forms a hydrogen bond with R-134 and close interactions with F-125 and T-123 (Fig. 2). R-134 and F-125 have previously been demonstrated to be the key residues involved in RNA binding [7]. In the present study, we found that both lead compounds form a hydrogen bond interaction with R-134 and interact with other key residues (Figs. 3 and 4) that can negatively influence the binding of RNA to VP40, potentially inhibiting the Ebola virus replication process. In support of the docking analysis results, molecular dynamics simulations showed that these two lead compounds are more stable and exhibit stronger binding to VP40 due to forming a greater number of hydrogen bonds. The MM-PBSA analysis also showed that these lead compounds displayed a high binding affinity throughout the simulation.

Finally, the molecular properties, carcinogenicity and oral toxicity (LD50) parameters of these compounds indicated that emodin-8-beta-D-glucoside might be a more promising lead candidate than tonkinochromane_G for the future development of an effective antiviral agent against the Ebola virus. It is also to be noted that emodin-8-O-beta-D-glucoside is extracted from the herb *Polygonum cuspidatum* Sieb. etZucc, which is used for the treatment against hepatitis and emodin-8-O-beta-D-glucoside itself, demonstrated pharmacological importance in neuro-protective effects against cerebral ischemia-reperfused injury and glutamate-induced neuronal damage [45]. While computations do not provide a complete replacement for experimental research, the relationship between computational and experimental approaches is very crucial process to guide the experimental biologist in screening and synthesize the compound in more rational and rapid way [39]. Hence, we hope that our computational findings will be crucial for experimental biologists to develop antiviral agents very quickly against the Ebola virus.

## Conclusion

Emodin-8-beta-D-glucoside and tonkinochromane_G are the lead candidates screened from the TCM database that is predicted to have potential antiviral activity against Ebola infection. Docking analysis revealed that these lead compounds interact with R-134 and F-125, which are the key residues for RNA binding. Further, molecular dynamics simulation results also validated the effective binding of these two compounds with the target. Finally, predicted oral toxicity and other physicochemical properties showed that emodin-8-beta-D-glucoside can be safer and efficient lead candidate for the development of antiviral therapeutics.

## Additional files

Additional file 1:
**Multilingual abstracts in the six official working languages of the United Nations.** (PDF 373 kb) Additional file 2: Table S1. List of top 15 compounds from TCM database. **Table S2.** Top ranking TCM lead compounds obtained using iScreen against VP40. **Table S3.** Comparison of binding free energies of lead compounds using Autodock. **Table S4.** Energetic contribution of lead compounds and RNA with VP40. **Table S5.** Comparison of interacting residues of lead compounds. **Table S6.** ADME and Drug-likeness analysis. **Table S7.** Toxicity risks of lead compounds predicted by OSIRIS property explorer. **Table S8.** Prediction of oral toxicity (LD50). **Figure S1.** Overall flow chart of the present study. **Figure S2.** Crucial Hydrogen bond interaction formed between RNA and VP40. **Figure S3.** Crucial Hydrogen bond interaction formed between compound1 and VP40. **Figure S4.** Crucial Hydrogen bond interaction formed between compund2 and VP40. (DOC 8072 kb)
