# Supplementary material for: Virtual screening of the inhibitors targeting at the viral protein 40 of Ebola virus
Source: Infect Dis Poverty. 2016 Feb 17;5:12. doi: 10.1186/s40249-016-0105-1 (PMC4757971; doi:10.1186/s40249-016-0105-1)
Supplement: Additional file 1: — Multilingual abstracts in the six official working languages of the United Nations. (PDF 373 kb) [file 40249_2016_105_MOESM1_ESM.pdf]

Translation of the abstract into the six official working languages of the United Nations

منهج فحص تقديري مبنى على المستقبلات لإكتشاف مثبطات محتملة لفيروس الإيبولا VP40

كارثيك ف، ناجاسندارام ن، جورج بريا دوس س، تشيرانجيب تشاكرابورتي، شياو نونغ تشو، جي مينغ ليو و تشو هاى لونج

الملخص

الخلفية: يتسبب فيروس الايبولا فى عدوى شديدة ومدمرة للبشر والرئيسيات الأخرى، يقوم الفيروس بترميز البروتين الفيروسي 40(VP40) ، وهو البروتين الذى يتم تمثيله بصورة عالية والمسئول عن تنظيم تجمع وإطلاق الجسيمات الفيروسية في الخلية المضيفة، وحيث أن هذا البروتين (VP40) يلعب دورا بارزا في دورة حياة فيروس الإيبولا فإنه يعتبر هدفا رئيسيا للعلاج بمضادات الفيروسات وعلى الرغم من ذلك لا يوجد حاليا أي عقار فعال أو معتمد من هيئة الغذاء والدواء لعلاج عدوى فيروس الإيبولا مما يدعوا للحاجة الملحة لتطوير مثبطات فعالة مضادة للفيروس ذات صفات سلامة جيدة في مدة قصيرة.

الأساليب: ولمعالجة هذه المسألة هدفت الدراسة الحالية الى فحص وتقييم كفاءة وفاعلية بعض المركبات الواعدة المرشحة للإستخدام ضد عدوى فيروس الإيبولا، في البداية تم تصفية هذه المركبات بناءا على مجموع وقوة الإرتباط بين مكوناتها وبعد ذلك تم معرفة قاعدة ليبنسكي الخماسية والخواص التشابهية الاخرى للعقار لضمان سلامة المركبات المرشحة كعلاج وفى النهاية تم إستخدام تقنية المحاكاة الديناميكية الجزيئية للتحقق من صحة المركب المرشح للإستخدام.

النتائج: كشفت النتائج أن مركب إيمودين-8-بيتا-د-جلوكوسيدي من قاعدة بيانات الطب الصيني التقليدي (TCMD) يعد مركبا واعدا لإستهداف فيروس الإيبولا من خلال تثبيط نشاط البروتين الفيروسي VP40 كما أظهر المركب أيضا خصائص دوائية جيدة.

الخلاصة: ستساهم هذه الدراسة بحد كبير في تطوير عوامل مضادة للفيروسات أكثر تنافسية وقوة ضد عدوى فيروس الإيبولا.

Translated from English version into Arabic by Mohamed R. Habib, through

CHINESE CENTER FOR DISEASE CONTROL AND PREVENTION
NATIONAL INSTITUTE OF PARASITIC DISEASES

以埃博拉病毒编码病毒蛋白 VP40 为标靶的抗病毒抑制剂的虚拟筛选

Karthick V, Nagasundaram N,George Priya Doss C, Chiranjib Chakraborty, Siva R, 周晓农，刘继明，吕爱平，张戈，祝海龙

摘要

引言：埃博拉病毒对人类和其他灵长类动物而言具有高致病性和破坏性。埃博拉病毒编码病毒蛋白 40（VP40）具有调控病毒颗粒的组装和释放作用，且在宿主细胞内有高表达。由于 VP40 在埃博拉病毒的生命周期中的重要作用，因此被认为是可用于抗病毒治疗的一个关键标靶。目前还没有经过 FDA 批准的治疗埃博拉病毒感染的药物，因此导致迫切需要开发有效且安全的抗病毒抑制剂。

方法：本研究的目的是筛选抗埃博拉病毒感染的有效的先导化合物。首先，使用分子对接方法对先导分子进行筛选。接着，基于利平斯基五规则和药物相似性指标对先导化合物的安全性进行评估。最后，利用分子动力学模拟技术对先导化合物进行进一步验证。

结果：研究结果表明，从中国中医药数据库（TCMD）筛选出来的大黄素 8-β-D-葡萄糖苷具有抑制埃博拉病毒编码蛋白 VP40 的功能，且显示出良好的药代动力学特性，可以作为一个候选化合物。

结论：本研究将有助于开发抗埃博拉病毒感染的抑制剂。

Translated from English version into Chinese by Zhu Hai-long

Tri virtuel basé sur les récepteurs pour la découverte d'inhibiteurs potentiels de la PV40 du virus Ebola

Karthick V, Nagasundaram N, GeorgePriya Doss C, Chiranjib Chakraborty, Xiao-nong Zhou, Ji-ming Liu, Hai-long Zhu

Résumé

Contexte: Le virus Ebola est hautement pathogène et destructeur pour les humains et les autres primates. Il encode la protéine virale 40 (PV40), qui est fortement exprimée et régule l'assemblage des particules virales dans la cellule hôte et leur libération hors de celle-ci. Rouage essentiel dans le cycle vital du virus Ebola, la PV40 est considérée comme une cible majeure du traitement antiviral. Il n'existe cependant, à l'heure actuelle, aucun médicament efficace ou autorisé par la FDA pour le traitement de l'infection par le virus Ébola, de sorte qu'il est urgent de développer rapidement des inhibiteurs antiviraux efficaces et sûrs.

Méthodes: Afin de répondre à ce besoin, la présente étude avait pour but d'identifier un candidat potentiel efficace pour le traitement de l'infection par le virus Ebola. Les molécules potentielles ont d'abord été filtrées en fonction de leur *docking score*. La règle de cinq de Lipinski et d'autres propriétés de probabilité de médicament ont ensuite été prédites afin de vérifier le profil d'innocuité des candidats potentiels. Enfin, des simulations dynamiques moléculaires ont été réalisées afin de valider le composé potentiel.

Résultats: Nos résultats ont révélé que l'émodine-8-bêta-d-glucoside de la Base de données de médecine chinoise traditionnelle (TCMD) constituait un candidat potentiel actif, qui vise le virus Ébola en inhibant l'activité de la VP40 et qui présente de bonnes propriétés pharmacocinétiques.

Conclusion : Le présent rapport va considérablement aider au développement des antiviraux les plus compétitifs et robustes pour combattre l'infection par le virus Ebola.

Translated from English version into French by Suzanne Assenat, through

TRANSLATORS
WITHOUT BORDERS

Метод виртуального скрининга на основе рецепторов для обнаружения потенциальных ингибиторов вируса Эбола VP40

Картик В., Нагасундарам Н., ДжорджПрия Досс К., Чиранджиб Чакраборти, Сяо-нун Чжоу, Цзи-мин Лю, Хай-лун Чжу

Аннотация

Предисловие: Вирус Эбола является высокопатогенным и разрушительным для человека и других приматов. Вирус Эбола кодирует вирусный белок 40 (VP 40), который является высоковыраженным и регулирует сборку и выпуск вирусных частиц в клетку-хозяин. Поскольку VP40 играет выдающуюся роль в жизненном цикле вируса Эбола, то он является ключевой целью антивирусного лечения. Однако, в настоящее время не имеется эффективного или одобренного FDA препарата для лечения инфекции вируса Эбола, что создает срочную необходимость разработки эффективных противовирусных ингибиторов, демонстрирующих хорошие профили безопасности, в короткой перспективе.

Методы: Чтобы решить эту проблему, настоящее исследование было направлено на скрининг эффективного ведущего кандидата против инфекции Эбола. Сначала, ведущие молекулы фильтровались на основе счета докингов. Затем, предсказываются правило пяти Липински и другие свойства подобия лекарству для обеспечения профиля безопасности

ведущих кандидатов. Наконец, инициировалась методика симуляций молекулярной динамики для оценки ведущего соединения.
Результаты: Наши результаты показали, что эмодин-8-бета-D-глюкозид из Базы данных традиционной китайской медицины (TCMD) представляет собой активный ведущий кандидат, который таргетирован на вирус Эбола, ингибируя активность VP40, и который проявляет хорошие фармакокинетические свойства.
Вывод: Настоящее исследование окажет значительную помощь в разработке наиболее конкурентоспособных и сильных антивирусных агентов против инфекции Эбола.

Translated from English version into Russian by Alexander Somin, through

TRANSLATORS WITHOUT BORDERS

Enfoque de cribado virtual basado en los receptores para el descubrimiento de inhibidores potenciales del virus de Ébola VP40

Karthick V, Nagasundaram N, GeorgePriya Doss C, Chiranjib Chakraborty, Xiao-nong Zhou, Ji-ming Liu, Hai-long Zhu

Sumario

Información de fondo: El virus del Ébola es altamente patógeno y destructivo para los seres humanos y otros primates. El virus del Ébola codifica la proteína viral 40 (VP40), que se expresa altamente y regula el ensamblaje y la liberación de las partículas virales en la célula anfitriona. Debido a que la VP40 desempeña un papel prominente en el ciclo de vida del virus del Ébola, se la considera objetivo clave para el tratamiento antiviral. Sin embargo, actualmente no existe un fármaco efectivo o aprobado por la FDA para el tratamiento de la infección por el virus del Ébola, lo que resulta en una necesidad urgente de desarrollar inhibidores antivirales eficaces que muestren buenos perfiles de seguridad en un corto período.
Métodos: Para abordar este problema, el presente estudio tuvo como objetivo detectar el fármaco potencial eficaz contra la infección del Ébola. Inicialmente, las moléculas de partida fueron filtradas en base a la puntuación de acoplamiento. Posteriormente, se predice que la regla de cinco de Lipinski y la propiedad de plausibilidad del otro fármaco garantizarán el perfil de seguridad de los fármacos potenciales. Finalmente, la técnica de simulaciones de dinámica molecular se inició para validar el compuesto potencial.
Resultados: Nuestros resultados revelaron que el emodin-8-b-D-glucósido de la base de datos de la Medicina Tradicional China (TCMD) representa un fármaco potencial activo que apunta al virus del Ébola mediante la inhibición de la actividad de la VP40, y que muestra buenas propiedades farmacocinéticas.
Conclusión: Este informe ayudará considerablemente en el desarrollo de los agentes antivirales más competitivos y sólidos contra la infección del Ébola.

Translated from English version into Spanish by María Diehn, through

TRANSLATORS WITHOUT BORDERS
