# Supplementary material for: Virtual screening of the inhibitors targeting at the viral protein 40 of Ebola virus
Source: Infect Dis Poverty. 2016 Feb 17;5:12. doi: 10.1186/s40249-016-0105-1 (PMC4757971; doi:10.1186/s40249-016-0105-1)
Supplement: Additional file 2: Table S1. — List of top 15 compounds from TCM database. Table S2. Top ranking TCM lead compounds obtained using iScreen against VP40. Table S3. Comparison of binding free energies of lead compounds using Autodock. Table S4. Energetic contribution of lead compounds and RNA with VP40. Table S5. Comparison of interacting residues of lead compounds. Table S6. ADME and Drug-likeness analysis. Table S7. Toxicity risks of lead compounds predicted by OSIRIS property explorer. Table S8. Prediction of oral toxicity (LD50). Figure S1. Overall flow chart of the present study. Figure S2. Crucial Hydrogen bond interaction formed between RNA and VP40. Figure S3. Crucial Hydrogen bond interaction formed between compound1 and VP40. Figure S4. Crucial Hydrogen bond interaction formed between compund2 and VP40. (DOC 8072 kb) [file 40249_2016_105_MOESM2_ESM.doc]

**Receptor-based virtual screening approach for the discovery of potential inhibitors of Ebola virus VP40**

Karthick. V, Nagasundaram. N,George Priya Doss. C, Chiranjib Chakraborty,

Xiaonong Zhou, Jiming Liu, Hailong Zhu

**Supplementary Table Information**

**Table S1** List of top 15 compounds from TCM database

**Table S2** Top ranking TCM lead compounds obtained using iScreen against VP40

**Table S3** Comparison of binding free energies of lead compounds using Autodock.

**Table S4** Energetic contribution of lead compounds and RNA with VP40

**Table S5** Comparison of interacting residues of lead compounds

**Table S6** ADME and Drug-likeness analysis

**Table S7** Toxicity risks of lead compounds predicted by OSIRIS property explorer

**Table S8** Prediction of oral toxicity (LD50)

**Supplementary Figure Information**

**Figure S1** Overall flow chart of the present study

**Figure S2** Crucial Hydrogen bond interaction formed between RNA and VP40

**Figure S3** Crucial Hydrogen bond interaction formed between compound1 and VP40

**Figure S4** Crucial Hydrogen bond interaction formed between compund2 and VP40

**Table S1** List of top 15 compounds and from TCM database

| **Compound name** | **Structure** | **Docking score** |
| --- | --- | --- |
| emodin-8-beta-Dglucoside | 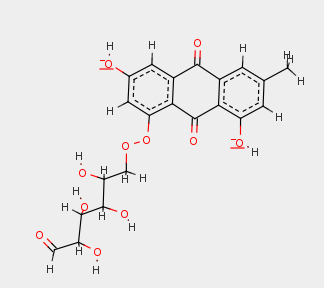 | -84.05 |
| tonkinochromane_G | 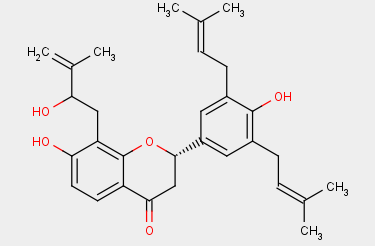 | -82.62 |
| neoglucobrassicin | 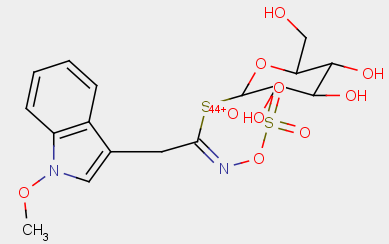 | -82.44 |
| glisoflavanone | 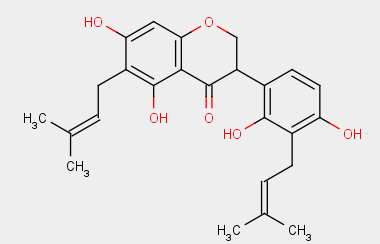 | -81.67 |
| rosmarinic_acid_ethyl_ester | 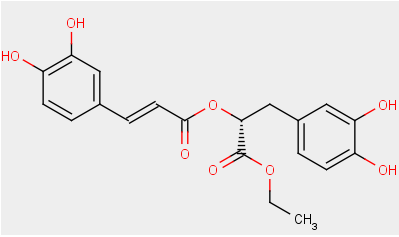 | -80.47 |
| 2-[(6Z.9Z_12Z)-heptadeca-6_9_12-trienyI]-6-hydroxybenzoic_acid | Not available | -80.40 |
| chrysophanol-8-beta-D-glucoside | 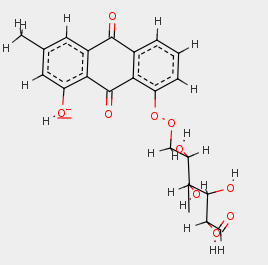 | -80.192 |
| 3_4-dihydro-3-methoxypaederoside | 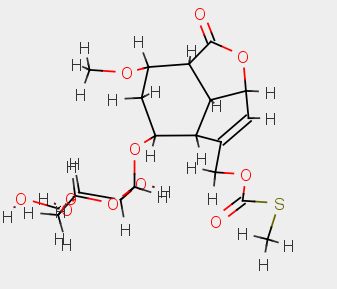 | -78.63 |
| Melittoside | 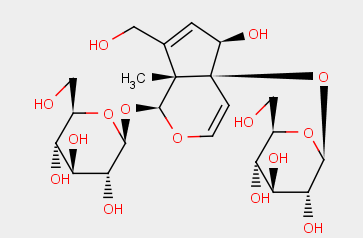 | -78.60 |
| beta-methoxylforsythoside | 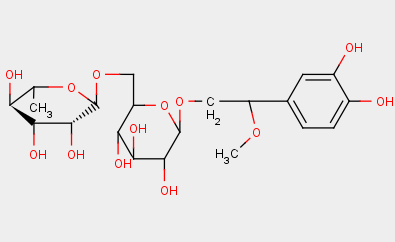 | -77.96 |
| glucobrassicin | 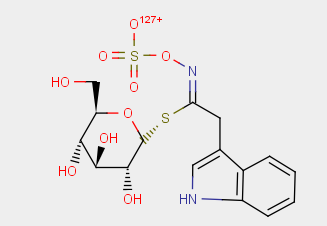 | -77.73 |
| manninotriose | 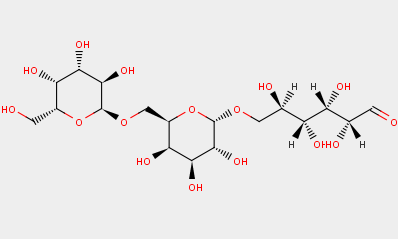 | -77.03 |
| D-mannitol_monohexadecanoate | 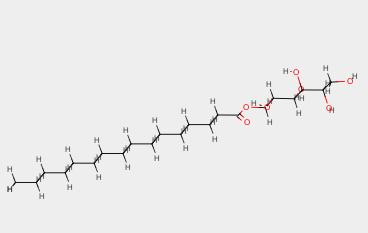 | -76.96 |
| 4__O-methyl_myricetin_3-O-(6-O-alpha-L-rhamnopyranosyl)-beta-D-glucopyranoside | 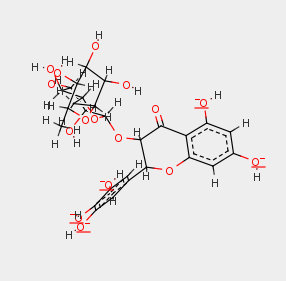 | -76.45 |
| (-)-epicatechin-3-O-gallate | 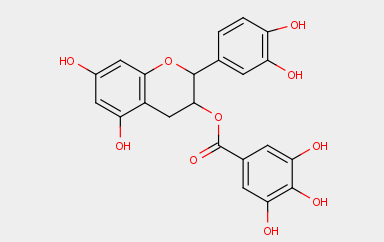 | -75.84 |

**Table S2** Top ranking TCM lead compounds obtained using iScreen against VP40

| **S. No** | **Ligand information** | **Score** | **Rank** |
| --- | --- | --- | --- |
| 1 | emodin-8-beta-Dglucoside (compound 1) | -84.05 | 1 |
| 2 | tonkinochromane_G (compound 2) | -82.62 | 2 |
| 3 | Neoglucobrassicin (compound 3) | -82.44 | 3 |

**Table S3** Comparison of binding free energies of lead compounds using Autodock.

| **S. No** | **Ligand information** | **Docking 1** | **Docking 2** | **Docking 3** | **Avg** |
| --- | --- | --- | --- | --- | --- |
| 1 | Compound 1 | -8.62 | -8.86 | -8.84 | -8.77 |
| 2 | Compound 2 | -8.84 | -8.46 | -8.92 | -8.74 |
| 3 | Compound 3 | -6.26 | -6.4 | -6.38 | -6.34 |

**Table S4 Energetic contribution of lead compounds and RNA with VP40**

| **Ligand information** | **Total Ligand‐Receptor Interaction Energy**  **(Kcal/mol)** | **Ligand‐Receptor Van der Waals Energy**  **(Kcal/mol)** | **Ligand‐Receptor Electrostatic Energy**  **(Kcal/mol)** | **Ligand‐Receptor Hydrogen Bond Energy**  **(Kcal/mol)** | **Ligand‐Receptor Solvation Free Energy**  **(Kcal/mol)** | **Ligand‐Receptor Conformational Entropy**  **(Kcal/mol)** |
| --- | --- | --- | --- | --- | --- | --- |
| Compound 1 | -10.57 | -8.62 | -0.32 | -1.20 | -1.08 | 0.65 |
| Compound 2 | -9.28 | -6.43 | -0.91 | -2.63 | 0.04 | 0.65 |

**Table S5** Comparison of interacting residues of lead compounds

| **Compound 1** | R-134, N-136, T-173, T-123, F-172, H-124, Y-171, F-125 |
| --- | --- |
| **Compound 2** | R-134, T-173, T-123, F-172, H-124, Q-170, Y-171, G-126, F-125 |

Red color indicates Hydrogen bond interactions

**Table S6** ADME and Drug-likeness analysis

| **Ligand information** | **MolLogP** | **Molecular weight** | **Hydrogen acceptors** | **Hydrogen donors** | **Drug-likeness score** |
| --- | --- | --- | --- | --- | --- |
| Compound 1 | -0.12 | 432.11 | 10 | 6 | 0.88 |
| Compound 2 | 7.13 | 476.26 | 5 | 3 | 0.36 |

Red color indicates violation

**Table S7** Toxicity risks of lead compounds predicted by OSIRIS property explorer

| **Compound** | **Mutagenic** | **Tumorigenic** | **Reproductive effect** | **Predicted LD50 (mg/kg)** | **Toxicity Class** |
| --- | --- | --- | --- | --- | --- |
| Compound 1 | No | No | No | 5000 | 5 |
| Compound 2 | No | No | No | 2000 | 4 |


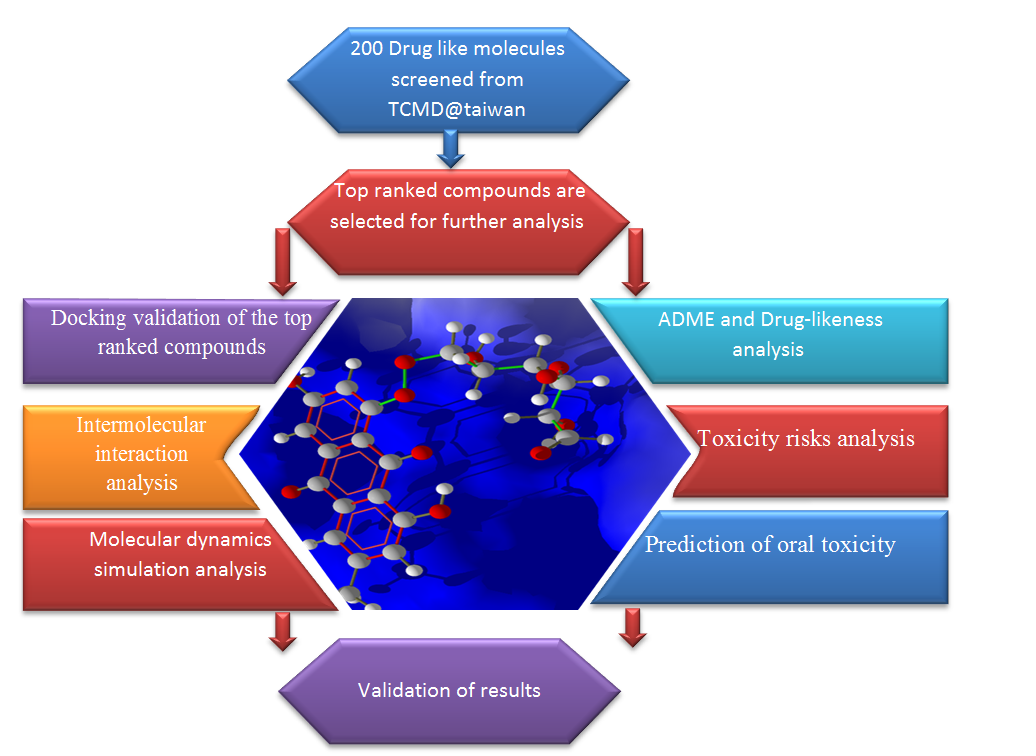


**Figure S1** Overall flow chart of the present study


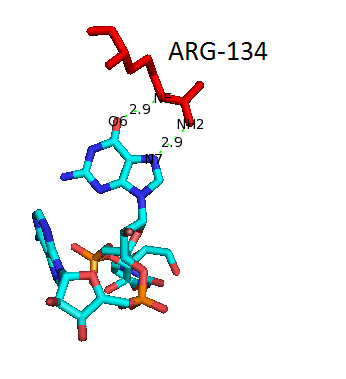


**Figure S2** Crucial Hydrogen bond interaction formed between RNA and VP40

**
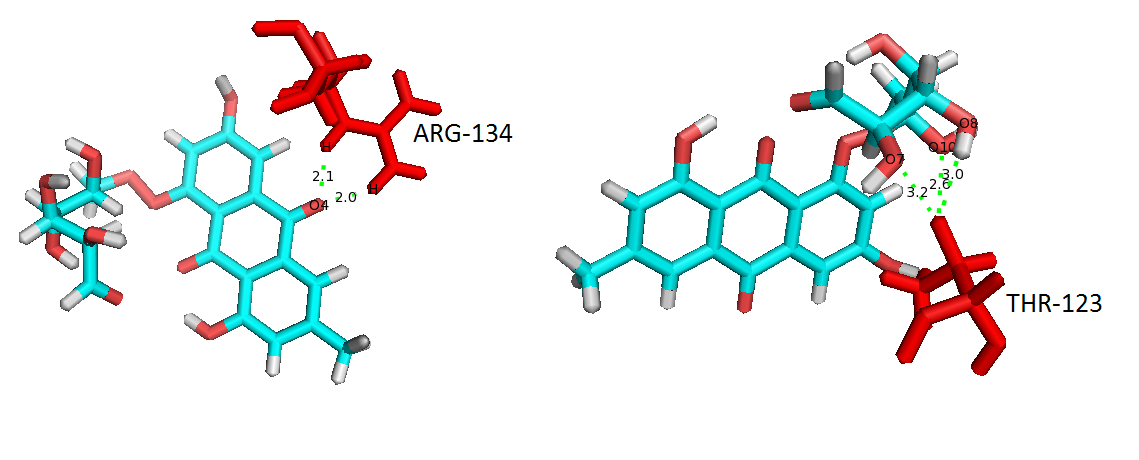
**

**Figure S3** Crucial Hydrogen bond interaction formed between compound1 and VP40


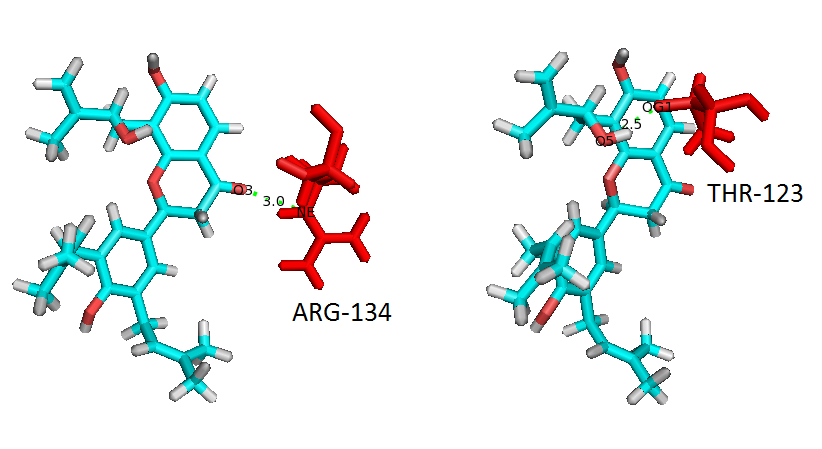


**Figure S4** Crucial Hydrogen bond interaction formed between compound2 and VP40
